# Horizontal and Multidirectional Single-Leg Hop Tests Predict Isokinetic Quadriceps and Hamstring Strengths After Anterior Cruciate Ligament Reconstruction

**DOI:** 10.1177/19417381261486269

**Published:** 2026-11-04

**Authors:** Ayşe Birsu Topcugil Kırık, Enes Akdemir, Emre Karaduman, Nizamettin Güzel, Ahmet Serhat Genç, Berna Anıl, Esra Korkmaz Salkılıç, Lokman Kehribar, Yılmaz Önder, Ali Kerim Yılmaz

**Affiliations:** Faculty of Medicine, Dokuz Eylül University, İzmir, Türkiye; Faculty of Yaşar Doğu Sports Sciences, Ondokuz Mayıs University, Samsun, Türkiye; Faculty of Yaşar Doğu Sports Sciences, Ondokuz Mayıs University, Samsun, Türkiye; Faculty of Medicine, Dokuz Eylül University, İzmir, Türkiye; Department of Orthopedics and Traumatology, Samsun Training and Research Hospital, Samsun, Türkiye; Faculty of Yaşar Doğu Sports Sciences, Ondokuz Mayıs University, Samsun, Türkiye; Faculty of Yaşar Doğu Sports Sciences, Ondokuz Mayıs University, Samsun, Türkiye; Faculty of Medicine, Dokuz Eylül University, İzmir, Türkiye; Department of Orthopaedics and Traumatology, Atatürk Training and Research Hospital, İzmir Katip Celebi University, İzmir, Türkiye; Faculty of Yaşar Doğu Sports Sciences, Ondokuz Mayıs University, Samsun, Türkiye

**Keywords:** anterior cruciate ligament reconstruction, hop test, isokinetic strength, knee function, rehabilitation

## Abstract

**Background:**

After anterior cruciate ligament reconstruction (ACLR), isokinetic strength and single-leg hop tests (SLHT) are used to assess return to sport readiness. This study examined the relationships and predictive effects of isokinetic knee extension (Ex) and flexion (Flx) strength with 5 SLHTs at 6 to 8 months post-ACLR.

**Hypothesis:**

There would be significant correlation between knee Ex and Flx strengths on the operated side and 5 different SLHTs; SLHTs would significantly predict knee isokinetic Ex and Flx strength.

**Study Design:**

Cross-sectional study.

**Level of Evidence:**

Level 3.

**Methods:**

Fifty-one male participants underwent unilateral ACLR. Lysholm, International Knee Documentation Committee (IKDC), and Tegner scales were assessed preoperatively and 6 to 8 months postoperatively. At the same follow-up, isokinetic strength at 60°/s, 180°/s, and 240°/s and 5 SLHTs were tested on both limbs. Data were analyzed with paired *t* tests, Pearson correlations, Fisher’s exact test, and simple linear regression; limb symmetry index (LSI) cut-off was ≥85%.

**Results:**

Isokinetic strength and single-leg hop (SH), crossover hop, and medial side triple-hop (TH) distances did not differ significantly between limbs (*P* > 0.05), whereas TH and 90° medial rotation hop distances were significantly shorter on the operated side (*P* < 0.05). Participants’ Lysholm and IKDC scores improved significantly at 6 to 8 months post-ACLR (*P* < 0.001), but Tegner activity level did not change significantly (*P* > 0.05). High correlation was found between Ex strength at 60°/s and SH and TH distances on the operated side, and high-moderate correlations were found at 180°/s and 240°/s. Flx strengths showed a similar but slightly lower degree of correlation. Simple linear regression models revealed that especially SH and TH distances significantly predicted both Ex and Flx strengths (adjusted *R*^2^ 0.26-0.52; *P* < 0.001). Among the LSI values, only a significant correlation was found between 60°/s Ex and TH LSI ratio (*P* = 0.01).

**Conclusion:**

The findings show that SH and TH tests were highly predictive of isokinetic knee strength after ACLR.

**Clinical Relevance:**

SLHTs after ACLR can be used as a practical and accessible complement to isokinetic measurements.

Anterior cruciate ligament (ACL) rupture causes knee joint instability and neuromuscular deficit, often leading to significant quadriceps and hamstring strength imbalances.^7,17,21,28^ Although ACL reconstruction (ACLR) aims to restore knee kinematics for return to sport (RTS), approximately 20% of patients suffer reinjuries.^4,40^ The risk of reinjury depends on many factors, and has been associated directly with muscle strength imbalances, losses in functional performance, and poor psychological readiness.^16^

After ACLR, quadriceps atrophy and hamstring weaknesses can negatively affect knee mechanics.^1,8,32,40,43^ Therefore, it is of great importance to evaluate both knee extension (Ex) and flexion (Flx) strength and function in RTS evaluations after ACLR. Although isokinetic assessments evaluate quadriceps and hamstring strengths most objectively, they are limited in that they are expensive, require expertise to assess, and do not fully reflect multijoint functionality.^44^ Consequently, single-leg hop tests (SLHTs) are used widely to provide functional outcomes, serving as a key component within the multifactorial decision-making process for RTS.^2,18,25,29,35^

The limb symmetry index (LSI) is used to assess the imbalance between the operated and nonoperated limbs; an LSI between 85% and 90% in all strength and hop tests indicates a safe RTS outcome.^42^ However, researchers have reported that traditionally applied forward straight hops are a limitation, especially in SLHTs,^10,12^ and emphasized the importance of multidirectional tests as well as forward tests, especially for RTS decision-making after ACLR.^15^ Indeed, studies have reported increased asymmetry rates in multidirectional tests compared with traditionally applied SLHTs.^15,25^

While previous studies have established significant correlations between isokinetic knee Ex, Flx strengths, and SLHT distances, this evidence remains insufficient.^33,36,44^ Previous analyses focused primarily on univariate correlations, which fail to capture the complex relationship between isolated joint strength and multijoint dynamic stability. Furthermore, comprehensive predictive models are scarce in the literature; only 1 study has performed simple regression analyses between post-ACLR isokinetic knee strengths and SLHT, and it was restricted solely to the forward single-leg hop for distance (SH) test.^40^ Thus, a significant knowledge gap exists regarding how isokinetic strength relates to, and predicts, performance across comprehensive, multidirectional functional movements.

Considering this knowledge gap, our study aims to investigate the relationships between knee isokinetic Ex and Flx strengths at 3 different angular velocities on the operated and nonoperated sides after ACLR and the jump distances in 5 different SLHTs. The study hypothesized that there would be significant correlations between the knee Ex and Flx strengths on the operated side and 5 different SLHTs and that SLHTs would significantly predict knee isokinetic Ex and Flx strengths.

## Methods

### Study Design

This cross-sectional study was approved by the Dokuz Eylül University Clinical Research Ethics Committee (approval number: 2025/24-10) and was conducted according to the Declaration of Helsinki. All participants were included in the study after obtaining their informed consent. Participant inclusion criteria were being between 18 and 44 years of age and having undergone unilateral ACLR using hamstring tendon autograft. Participants who had a previous contralateral knee injury, had a concomitant meniscal injury, had reconstruction of other ligaments of the knee in addition to the ACL, did not participate regularly in the rehabilitation program, and were unable to perform physical tests were excluded from the study. In this study, the Lysholm scale was used to assess signs of instability in the knee and other sites, the Tegner scale to determine eligibility for regular physical activity or RTS, and the International Knee Documentation Committee (IKDC) scale to measure improvement in function, symptoms, and RTS. Preoperataive data were recorded on all scales. Lysholm, Tegner, and IKDC scores were then obtained again after ACLR (6 to 8 months postoperatively). For knee strength assessment, isokinetic knee strength and SLHTs were evaluated on the operated and nonoperated sides. All postoperative assessments ended with 3 laboratory visits. At the first visit, the postoperative scale scores of the participants were recorded, and a familiarization session was performed. On the other 2 visits, participants completed 1 of the 2 performance tests in a randomized order. A 48-hour rest was given between visits.

### Participants

The study included 51 recreationally active men who underwent ACLR with unilateral hamstring tendon autograft by the same surgeon. An a priori power analysis was conducted (G*Power software, Version 3.1) to determine the required sample size using a moderate-to-large effect size (*f*^ 2^ = 0.25), an alpha level of 0.05, and a statistical power of 0.80. The analysis indicated that a minimum of 34 participants was required for a simple linear regression model with a single explanatory variable; thus, our sample of 51 was deemed highly adequate. Evaluations were performed 6 to 8 months postoperatively after the completion of the rehabilitation process (Figure 1). All participants were referred to the same physiotherapist to standardize their postoperative rehabilitation programs. Also, the choice of a homogeneous participant group in the study contributed to determining whether SLHTs can be used to predict knee Ex and Flx strengths in ACLR.

**Figure 1.**
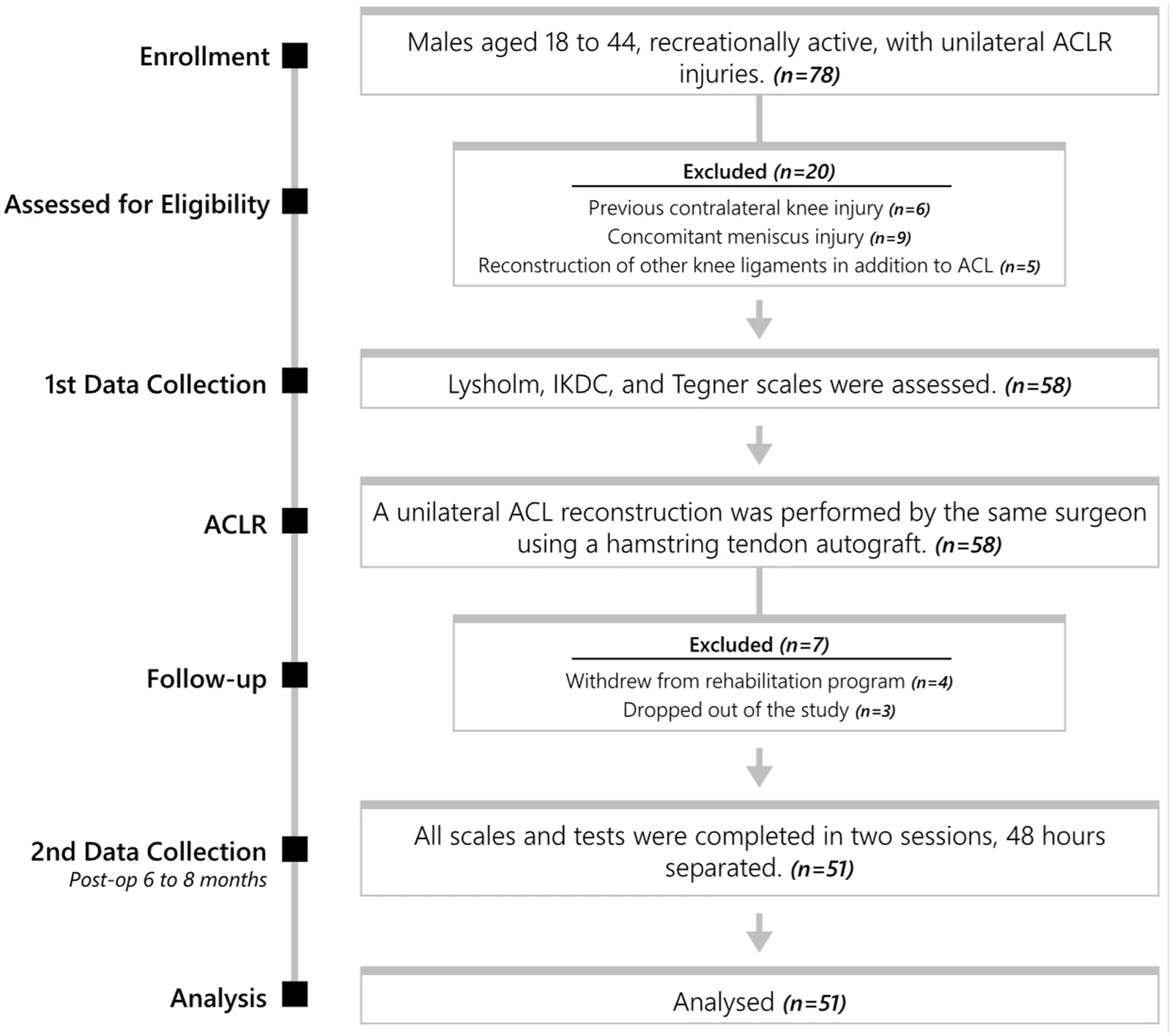
Study flowchart. ACL, anterior cruciate ligament; ACLR, ACL reconstruction; IKDC, International Knee Documentation Committee.

### Surgical Technique

Patients underwent ACLR with unilateral hamstring tendon autograft (Modified All-Inside). Anatomical single-bundle reconstruction was achieved with a quadruple-folded semitendinosus autograft. Femoral fixation was performed with a fixed-loop button system and tibial fixation with an adjustable loop cortical sling device. Femoral tunnels were drilled via the anteromedial portal, and tibial tunnels were created anatomically. The graft was tensioned at 20° of knee Flx and cycled through Flx and Ex to ensure appropriate tension and placement. This standardized surgical procedure minimized variability across participants.

### Test Procedures

#### Single-Leg Hop Tests

SLHTs were performed on a 5-cm wide and 6-meter length test band to assess hop distance using the SH, triple hop for distance (TH), crossover hop for distance (CH), medial side triple hop for distance (MSTH), and 90° medial rotation hop for distance (MRH) protocols, respectively. A 30-cm length reference band was placed at the starting point of the 6-meter test band common to all tests, and this area was considered the measurement baseline. The test procedure was explained to the participants in detail, both through verbal explanation and practical demonstration. Three repetitions were performed for each test and lower extremity. The jump distance was measured from the starting line to the point where the subject’s heel made contact, and the longest distance (in centimeters) was recorded. During the test, participants were allowed to use their upper limbs freely. To achieve a valid measurement, the tested lower extremity had to end up on the test band after the jump. If balance was not maintained during the test, if the tested limb did not end on the test band, or if additional jumps were performed, the relevant trial was considered invalid and repeated.

#### Isokinetic Strength Measurement

Participants’ lower extremity isokinetic muscle strength was assessed using a computer-controlled isokinetic dynamometer (Humac Norm Testing and Rehabilitation System, CSMI). After a standardized warm-up, the dynamometer settings were adjusted according to the standard knee Ex and Flx testing protocol. Knee range of motion (ROM) was set between 0° and 90°, with the hip joint positioned at 85° (0° = full Ex). The trunk, pelvis, and tested limb were stabilized using straps, participants held the side hand grips, and the nontest limb was restrained to minimize compensatory movements. Gravity correction was applied by determining the passive torque at 90° before testing. Isokinetic testing was conducted in concentric/concentric mode at angular velocities of 60°/s (4 familiarization trials + 5 test repetitions), 180°/s (4 + 5), and 240°/s (4 + 15), in accordance with the manufacturer’s protocol. A 30-second rest period was provided between velocities and 15 seconds between familiarization and test sets. Verbal instructions and strong encouragement were given throughout testing. Peak torque values (in Newton-meters) were recorded.

#### Lysholm Knee Scoring Scale

The Lysholm knee score is a case-specific outcome assessment measure consisting of 8 domains. The test is scored between 0 and 100 points, with a score of 95 to 100 being a perfect score.

#### International Knee Documentation Committee

The IKDC subjective score consists of 18 questions assessing symptoms, sporting activities, and functional performance. It comprehensively reflects the participant’s health status and includes questions about simple daily activities such as the use of support, feeling of locking in the knee, and climbing stairs.

#### Tegner Activity Scale

The Tegner Activity Scale was designed as a scale that assesses the activity level in patients with ligament injury from 0 to 10 to complement the Lysholm knee score.

### Statistical Analysis

All statistical analyses were processed using the SPSS software Version 25 (IBM). Descriptive data were presented as means and standard deviations, median, minimum, and maximum. The normality of each variable’s distribution was determined by a histogram and the Kolmogorov-Smirnov normality test. Paired sample *t* tests were used to determine the differences between the surgical and nonsurgical sides in isokinetic knee strength and SLHTs, as well as to analyze the differences between pre- and postoperative patient-reported outcomes. Furthermore, LSI values were divided into 2 groups, ≥85% and <85%, for isokinetic knee strength and SLHTs, and the relationship between the LSI of Ex torque and the LSI of SLHTs was analyzed using Fisher’s exact test. Pearson’s correlation coefficient (*r*) was used to examine correlations between isokinetic knee strength and SLHTs. Correlations were classified as follows: trivial (<0.10), small (0.11-0.30), moderate (0.31-0.50), high (0.51-0.70), very high (0.71-0.90), and almost perfect (0.91-1.00).^22^ Separate linear regression analyses were performed to examine whether each hop test (SH, TH, CH, MSTH, and MRH) predicted isokinetic Ex and Flx peak torque individually at 60°/s, 180°/s, and 240°/s. Age was initially evaluated as a potential covariate using a stepwise selection procedure but was not retained in the final regression models because it did not contribute significantly. Regression assumptions, including linearity, normality of residuals, homoscedasticity, and independence of errors (Durbin-Watson statistic), were assessed through inspection of residual plots and diagnostic statistics before model interpretation. Because each final regression model included only a single predictor variable, multicollinearity diagnostics among hop-test variables were not applicable, as hop-test measures were not entered simultaneously into the same regression model. The coefficient of determination (*R*^2^) revealed the percentage of variability of the data explained by the adapted linear model. No formal adjustment for multiple comparisons was performed because the primary statistical analyses were planned a priori to address distinct research hypotheses rather than exploratory analyses of the same outcome. Accordingly, each analysis was interpreted independently, and a global correction (e.g., Bonferroni) was not applied because it may be overly conservative for hypothesis-driven analyses and increase the risk of Type II error. The statistical significance was based on a *P* value of <0.05.

## Results

Baseline characteristics are shown in Table 1.

**Table 1.** Baseline characteristics of participants

	Mean ± SD	Median (minimum-maximum)
Age, years	25.6 ± 5.7	25 (18-44)
Height, cm	177.8 ± 6.5	176 (167-192)
Weight, kg	82.4 ± 10.1	80 (67-105)
Body mass index, kg/m^2^	26.1 ± 3.0	26 (20-34)
Operative leg, n (%)		
Right	30 (58.8)	
Left	21 (41.2)	

There was no statistically significant difference between the Ex, Flx, and hamstring/quadriceps (H/Q) performance of surgical and nonsurgical legs. Similarly, the SH, CH, and MSTH distances between surgical and nonsurgical sides were not statistically significant (*P* > 0.05). However, the TH and MRH test distances showed slight differences between the sides (*P* < 0.05). Furthermore, the isokinetic knee strength (60°/s, 180°/s, and 240°/s) and SLHTs (SL, TH, CH, MSTH, and MRH) LSI means were ≥85% cutoff value (Table 2).

**Table 2:** Differences between-side comparison for isokinetic knee strength and SLHTs

	Surgical side	Nonsurgical side	*P*	LSI, %
Ex, N·m				
60°/s	188.5 ± 35.0	193.9 ± 49.7	0.36	101.9 ± 24.9
180°/s	121.0 ± 29.3	128.1 ± 29.1	0.07	97.2 ± 22.9
240°/s	105.8 ± 22.5	107.7 ± 25.7	0.46	100.8 ± 19.8
Flx, N·m				
60°/s	113.8 ± 29.4	117.2 ± 42.0	0.35	102.8 ± 26.0
180°/s	83.7 ± 24.4	85.1 ± 26.8	0.53	100.7 ± 20.4
240°/s	73.3 ± 18.8	74.5 ± 23.0	0.51	101.4 ± 18.8
H/Q ratio, %				
60°/s	60.6 ± 11.4	60.5 ± 14.5	0.98	
180°/s	70.3 ± 17.1	66.9 ± 14.7	0.31	
240°/s	69.8 ± 13.0	69.5 ± 13.4	0.87	
SLHT, cm				
SH	121.1 ± 15.7	124.0 ± 17.7	0.30	99.3 ± 17.9
TH	395.3 ± 61.7	433.8 ± 63.1	<0.001*	92.0 ± 13.4
CH	336.6 ± 55.6	355.8 ± 81.0	0.07	99.3 ± 30.2
MSTH	327.2 ± 55.3	339.5 ± 56.3	0.14	97.9 ± 17.9
MRH	105.4 ± 20.0	115.0 ± 21.7	0.005*	93.8 ± 19.9

Data are presented as mean ± SD. LSI was calculated as (surgical side/nonsurgical side) × 100. N·m, Newton-meter; CH, crossover hop; Ex, extension; Flx, flexion; H/Q, hamstring/quadriceps; LSI, limb symmetry index; MRH, medial rotation (90°) hop; MSTH, medial side triple hop; SH, single hop; SLHT, single-leg hop test; TH, triple hop.

* Statistically significant difference between groups (*P* < 0.05).

Athletes demonstrated significant postoperative improvements in Lysholm and IKDC scores (*P* < 0.001); however, no significant change was observed in Tegner activity scores (*P* > 0.05) (Figure 2).

**Figure 2.**
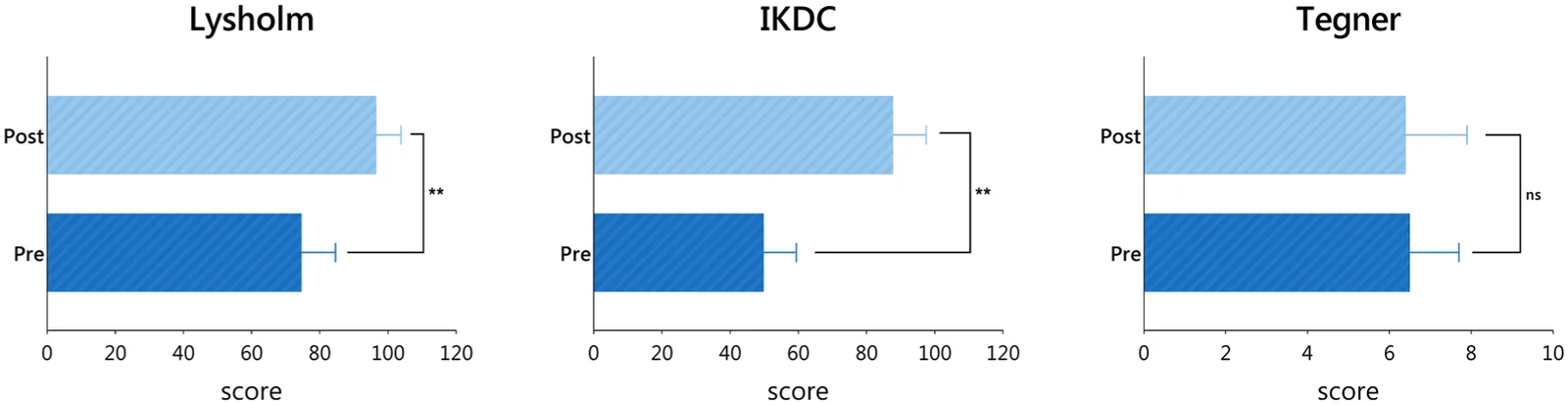
Pre- and postoperative comparison of differences in patient-reported outcomes. **P < 0.001. Data are presented as means and standard deviations. IKDC, International Knee Document Committee; Ns, nonsignificant.

Pearson correlation coefficients for each variable on the surgical side are presented in Figure 3. There were high-to-very-high positive correlations between 60°/s Ex isokinetic knee strength and SH (*r* = 0.730; *P* < 0.001) and TH (*r* = 0.654; *P* < 0.001). 60°/s Ex showed a moderate correlation with CH (*r* = 0.391; *P* = 0.004) and MSTH (*r* = 0.440; *P* = 0.001). However, there were moderate-to-high positive correlations between 180°/s Ex isokinetic knee strength and SH (*r* = 0.617; *P* < 0.001), TH (*r* = 0.619; *P* < 0.001), CH (*r* = 0.510; *P* < 0.001), and MSTH (*r* = 0.479; *P* < 0.001). There were moderate-to-high positive correlations between 240°/s Ex and SH (*r* = 0.588; *P* < 0.001), TH (*r* = 0.602; *P* < 0.001), CH (*r* = 0.528; *P* < 0.001), and MSTH (*r* = 0.484; *P* < 0.001). Furthermore, there was a moderate-to-high positive correlation between 60°/s, 180°/s, and 240°/s Flx isokinetic knee strength and SLHTs (*P* < 0.05), excluding CH (*P* > 0.05) (Figure 3).

**Figure 3.**
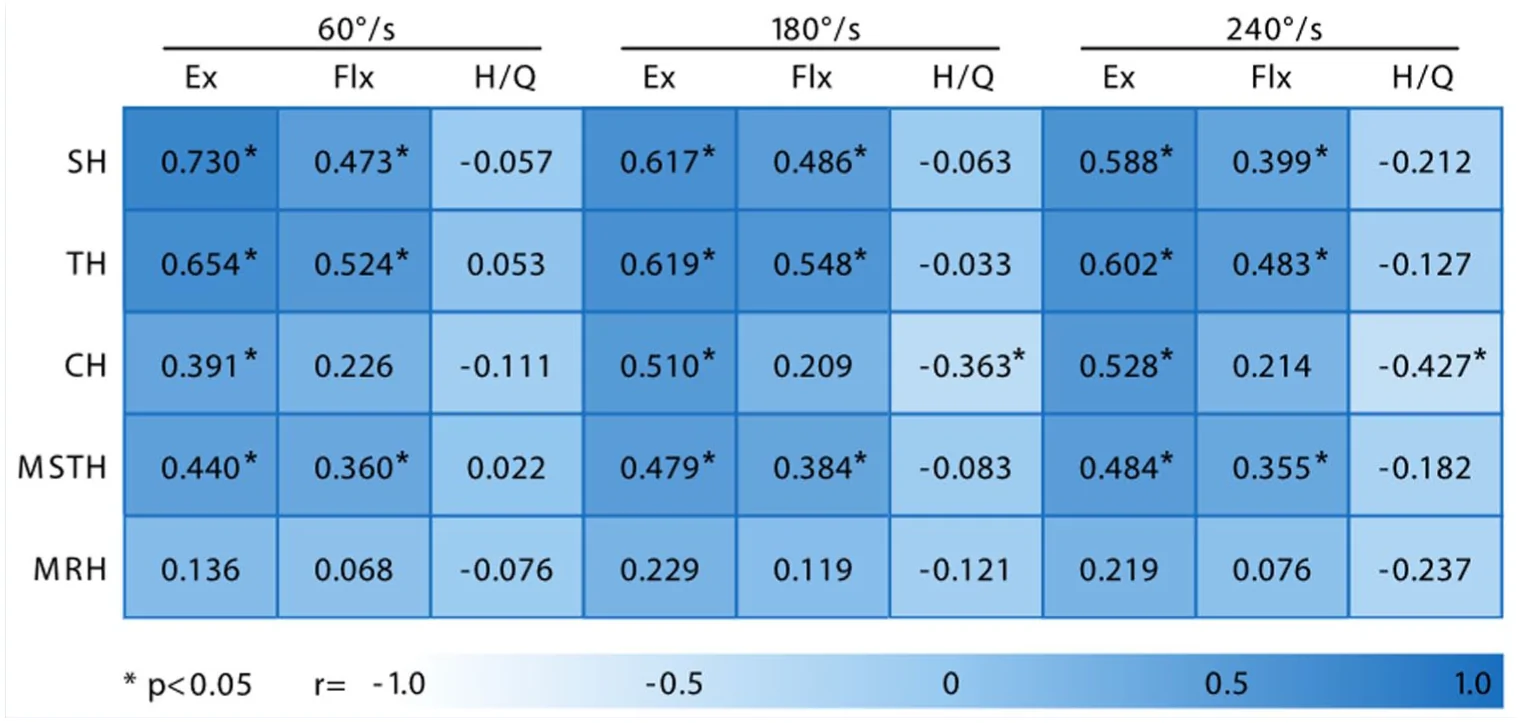
Correlation coefficients between isokinetic knee strength and SLHTs for the surgical side. The values in the boxes represent correlation coefficients (*r *) with *P* values <0.05. Ex, extension; Flx, flexion; H/Q, hamstring/quadriceps; SLHT, single-leg hop test.

Figure 4 displays the linear regression analysis results between isokinetic knee strength (Ex and Flx) at 60°/s, 180°/s, and 240°/s and SLHTs for the surgical side. SLHTs had a significant effect on isokinetic knee strength at 60°/s, 180°/s, and 240°/s Ex and Flx (*P* < 0.001). However, age was not a predictor variable for isokinetic knee strength on the surgical side (*P* > 0.05). The prediction model indicated that SH (adjusted *R*^2^ = 0.523, B = 1.629 [95% CI, 1.191-2.068]; *P* < 0.001) and TH (adjusted *R*^2^ = 0.416, B = 0.371 [95% CI, 0.248-0.495]; *P* < 0.001) are significant predictors for 60°/s Ex and best explain the isokinetic knee strength results among the analyzed outcomes. In addition, the SH (adjusted *R*^2^ = 0.367, B = 1.150 [95% CI, 0.728-1.571]; *P* < 0.001) and TH (adjusted *R*^2^ = 0.371, B = 0.294 [95% CI, 0.187-0.401]; *P* < 0.001) variables were significant predictors of isokinetic knee strength at 180°/s Ex in the surgical side. Similarly, we found that the SH (adjusted *R*^2^ = 0.332, B = 0.844 [95% CI, 0.510-1.177]; *P* < 0.001) and TH (adjusted *R*^2^ = 0.349, B = 0.220 [95% CI, 0.136-0.304]; *P* < 0.001) variables were significant predictors for 240°/s Ex and best explained the isokinetic knee strength results in the surgical side. Furthermore, the prediction model indicated that THs (adjusted *R*^2^ = 0.260, B = 0.250 [95% CI, 0.133-0.366]; *P* < 0.001) are significant predictors for 60°/s Flx and best explain the isokinetic knee strength results among the analyzed outcomes. In addition, the TH (adjusted *R*^2^ = 0.287, B = 0.217 [95% CI, 0.122-0.312]; *P* < 0.001) variables were significant predictors of isokinetic knee strength at 180°/s Flx in the surgical side. Similarly, the TH (adjusted *R*^2^ = 0.218, B = 0.147 [95% CI, 0.071-0.224]; *P* < 0.001) variables were significant predictors of isokinetic knee strength at 240°/s Flx in the surgical side.

**Figure 4.**
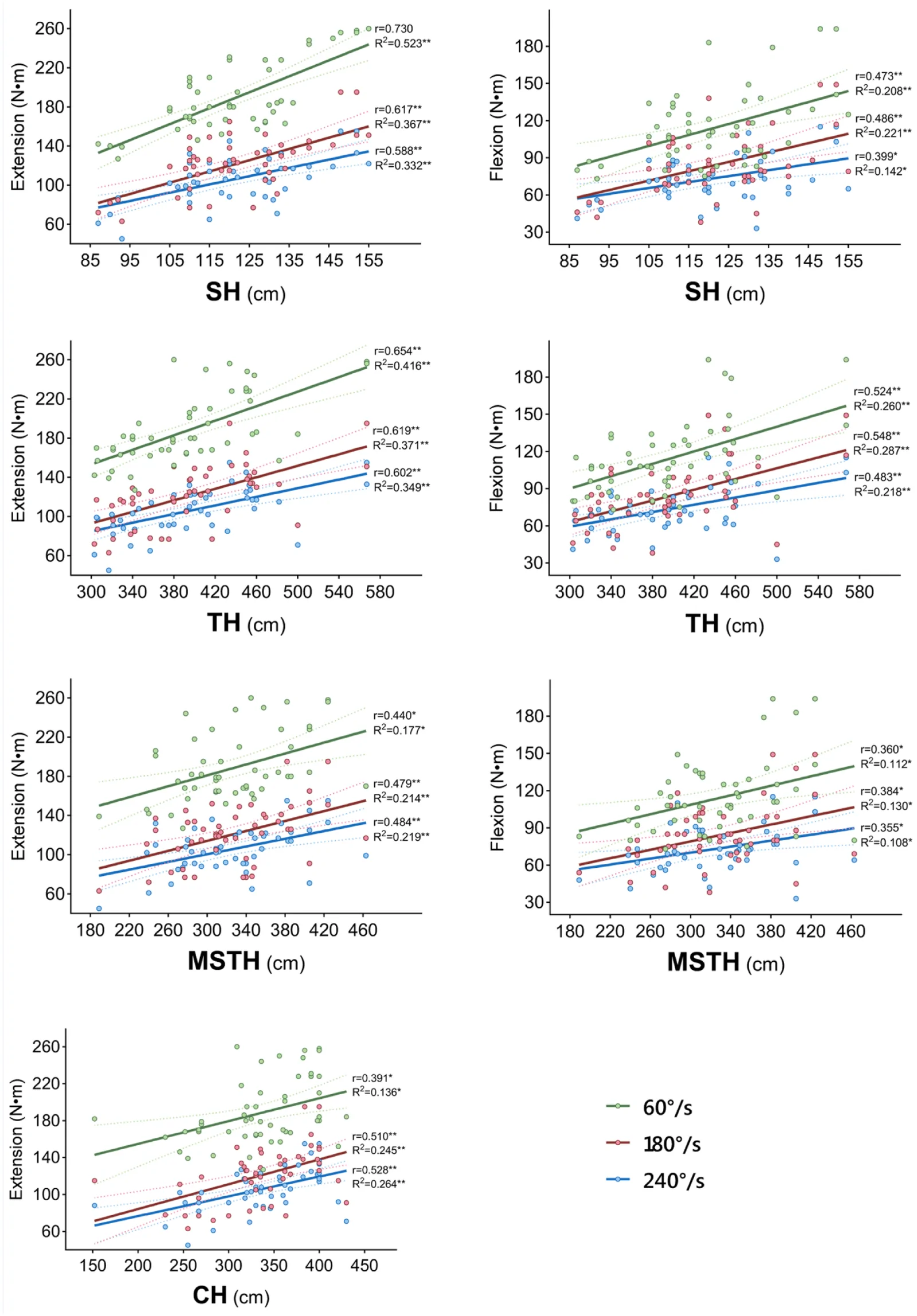
Scatterplots showing the relationship between isokinetic knee strength (Extension and Flexion) and SLHTs for the surgical side. Each point represents a single participant. The black lines indicate the best-fit line, with dotted lines representing the 95% CIs. Correlation (*r*) and regression (adjusted *R*^ 2^) coefficients between the SH, TH, MSTH, and CH tests, and 60°/s, 180°/s, and 240°/s isokinetic knee strength are displayed at the end of the best-fit line. N·m, Newton-meter; CH, crossover hop; MSTH, medial side triple hop; SH, single hop; TH, triple hop.*P < 0.05, **P < 0.001.

The results of Fisher’s exact test are shown in Table 3. The analysis revealed no significant relationships among the variables (*P* > 0.05), except between Ex 60°/s and TH of LSI values (*P* = 0.01), with a cutoff value of 85%.

**Table 3.** Relationship between LSI of isokinetic knee strength (Ex) and SLHTs

LSI, %		Ex 60°/s			Ex 180°/s			Ex 240°/s		
		≥85	<85	*P*	≥85	<85	*P*	≥85	<85	*P*
SL	≥85	35 (78%)	10 (22%)	0.62	34 (76%)	11 (24%)	0.32	35 (78%)	10 (22%)	>0.99
	<85	4 (67%)	2 (33%)		6 (100%)	0 (0%)		5 (83%)	1 (17%)	
TH	≥85	32 (86%)	5 (14%)	0.01*	30 (81%)	7 (19%)	0.47	29 (78%)	8 (22%)	>0.99
	<85	7 (50%)	7 (50%)		10 (71%)	4 (29%)		11 (79%)	3 (21%)	
CH	≥85	30 (77%)	9 (23%)	>0.99	30 (77%)	9 (23%)	>0.99	31 (79%)	8 (21%)	0.71
	<85	9 (75%)	3 (25%)		10 (83%)	2 (17%)		9 (75%)	3 (25%)	
MSTH	≥85	30 (71%)	12 (29%)	0.09	33 (79%)	9 (21%)	>0.99	33 (79%)	9 (21%)	>0.99
	<85	9 (100%)	0 (0%)		7 (78%)	2 (22%)		7 (78%)	2 (22%)	
MRH	≥85	30 (79%)	8 (21%)	0.47	32 (84%)	6 (16%)	0.12	31 (82%)	7 (18%)	0.44
	<85	9 (69%)	4 (31%)		8 (62%)	5 (38%)		9 (69%)	4 (31%)	

Values are given as n (%). LSI was calculated as (surgical side/nonsurgical side) × 100. CH, crossover hop; Ex, extension; LSI, limb symmetry index; MRH, medial rotation (90°) hop; MSTH, medial side triple hop; SH, single hop; TH, triple hop.

* Statistically significant difference (*P* < 0.05).

## Discussion

This cross-sectional study examined the associations between isokinetic knee strength data obtained at 6 to 8 months postoperatively and SLHT outcomes in male patients undergoing ACLR using unilateral hamstring tendon autograft. The major findings of the study showed that SH, TH, and MSTH were significant indicators of isokinetic Ex and Flx isokinetic strengths at different angular velocities, whereas CH was significant only in the Ex phase. In addition, there were moderate-to-high variable correlations between SLHT and isokinetic test results. All SLHT results showed higher correlations with the data obtained in the Ex phase compared with the Flx phase.

The importance of strength tests in the evaluation of the rehabilitation process after ACLR is frequently emphasized in the literature.^37^ In this context, although isokinetic tests are considered the gold standard,^14,34^ current approaches suggest that functional hop tests also play an important role in the assessment of functional capacity after ACLR.^9,23,35^ Therefore, current studies focus on more holistic evaluation approaches that include functional test results, rather than being limited to isolated isokinetic tests.^11,13,26,27,30,31,40,41^ Similar to previous studies that reported moderate to highly variable correlations between knee Ex strength and SLHT distances,^3,13,30,31,40^ the present study also found moderate to high correlations (*r* = 0.36-0.73) between SLHT distances and isokinetic strength results. In particular, SH and TH test distances were highly correlated (*r* = 0.59-0.73) with knee Ex strength at different angular velocities. These results are consistent with a previous study highlighting the explanatory potential of SLHT distance for knee Ex strength.^13^ However, contrary evidence indicates that there was no correlation between SH distance and knee Ex strength 6 and 12 months after ACLR and that SH cannot be used as a substitute for isokinetic knee Ex strength. In addition to the general trend in the literature, the correlations of multidirectional MSTH and MRA tests with isokinetic strength were also examined in our study. Accordingly, the correlations between the multidirectional MSTH and MRA tests and isokinetic strengths were relatively low compared with the forward directional SH, TH, and CH. In our study, the relationships between SLHTs and isokinetic evaluations were evaluated using simple linear regression models in addition to correlation. In this direction, it was determined that SH and TH were significant indicators for strength values in all angular velocities and phases (*P* < 0.001). However, recent evidence using similar regression models, the associations between the tests were not significant (*P* > 0.05).^40^ It is likely that various factors, such as angular velocity and follow-up time, especially the graft type, contribute to the contradictory results in both correlation analyses and regression models in the literature. These factors could also influence the relationships between isokinetic strength phases and angular velocities.

Although some studies emphasized that hop tests have higher correlations with isokinetic Ex strength compared with Flx strength,^3,13^ contrary evidence indicates that SH distance has a higher correlation with 180°/s isokinetic Flx strength.^38^ At the same angular velocity, high correlations have also been observed for both phases, although higher with Ex.^3^ In our study, isokinetic Ex strengths at all angular velocities had significantly higher correlations than Flx strengths, especially with SH. Notably, in patient cohorts where the majority (66%) undergo reconstruction using a patellar tendon graft, Flx strength at 180°/s angular velocity has shown higher correlations with SH compared with Ex strength.^38^ The reported optimal H/Q ratio for isokinetic knee strength is between 50% and 80%, although it increases depending on angular velocities.^45^ Therefore, the more dominant role of Ex strength in terms of jump biomechanics may be an important factor in the emergence of higher correlations compared with the Flx phase. However, the fact that the Flx phase had higher or similar correlations compared with the Ex phase may indicate that the study group had higher quadriceps atrophy compared to the hamstring.^24^ Furthermore, higher angular velocities appear to strengthen these relationships regardless of the graft type. Evidence indicates that higher correlations with SH for Ex strength occur at 180°/s compared with 60°/s, both in populations predominantly (69%) receiving patellar tendon grafts (where only Ex strengths were evaluated) and in those exclusively utilizing hamstring grafts.^3^ In our current study, in which all patients had undergone reconstruction with a hamstring graft, the correlations between SH and TH and isokinetic strengths increased with angular velocity.

In addition to the associations of strength-based parameters in isokinetic and SLHTs, the evaluation of LSIs is extremely important in making effective evaluations after ACLR.^23^ In particular, the commonly used LSI criterion for RTS reflects rates of 85% or higher.^35^ However, evidence indicates that an LSI of <85% can persist for up to 2 years after ACLR.^6,19,39^ Therefore, it is important to consider many different factors, such as the physical activity level, age, and rehabilitation process of the patient group in the evaluation of this criterion. LSI relationships in isokinetic Ex and SLHTs were analyzed using an 85% LSI cut-off value in our study. The analysis revealed a significant correlation only between TH and LSI rates at 60°/s (*P* = 0.01). Previous logistic regression models examining the LSI ratios of isokinetic Ex strength and SH have correctly classified participants who did not meet the 85% cut-off value for SH, while incorrectly classifying most of those who met the 85% cut-off value for Ex strength.^40^ These results reveal that, although the LSI of the SH may be useful in determining strength asymmetry and encouraging a focus on knee Ex strength, an LSI of ≥85% alone may not be sufficient to reflect Ex strength-related asymmetries.^40^ Supporting this finding, assessments in professional soccer players after ACLR demonstrated that >80% of the players did not exceed the 90% LSI criterion for isokinetic strength tests, even though 75% of the cohort exceeded the 90% LSI criterion in SLHT.^20^ Similarly, achieving an LSI of ≥85% for SH does not necessarily indicate a satisfactory improvement in knee Ex strength at the same rate.^5^ This discrepancy likely occurs because satisfactory LSI levels observed in functional tests are achieved when quadriceps deficiencies are compensated by physiological factors such as hip and trunk muscle strength and lower extremity neuromuscular control capacity.^5,46^

In addition to the general trend in the literature, this study is important in terms of including multidimensional tests such as MSTH and MRH in addition to the traditional SH, TH, and CH tests. Although the results suggest that forward hop tests may be correlated more highly with isokinetic strength, more comprehensive study designs are needed for these tests. Also, another strength of this study is that the findings reveal that SH and TH tests, in particular, can be important indicators for Ex and Flx strengths at different angular velocities.

### Limitations

This study has some important limitations. The fact that the patients in the study were between the ages of 18 to 44 years may be an important factor in terms of the emergence of age-related inter-participant differences. In addition to the variable age range, the fact that the study group consisted only of men and patients who underwent hamstring autograft is an important limitation in terms of the generalizability of the study results. Another important limitation is that only the postoperative results at 6 to 8 months were evaluated. Furthermore, although the patients were referred to the same physiotherapist, differences in the rehabilitation program may have affected the generalizability of the results. Moreover, given the cross-sectional design of this study, it should be noted that the regression models and associated terminology denote statistical variance explanation rather than temporal or causal relationships. Although the exclusion criteria in the study were important for ensuring group homogeneity, they also led to a relative limitation in the number of participants. Although our a priori power analysis confirmed the statistical adequacy of the current sample size, future studies with larger cohorts would be desirable to further strengthen the generalizability of the models.

## Conclusion

In postoperative strength evaluations, especially advanced functional tests such as SH and TH were important indicators for isokinetic strength results. However, the correlations between isokinetic tests for LSI ratios and functional performance tests were weak, indicating that functional symmetry alone may not fully reflect isolated strength recovery. Therefore, this study concludes that RTS decisions require a comprehensive evaluation combining both isokinetic and multidirectional functional tests. While future studies with higher sample sizes and with longer follow-up will contribute to a more comprehensive understanding of demographic and surgical factors, the current findings highlight that examining the relationships of isokinetic Ex and Flx strengths with alternative functional tests is highly valuable in terms of revealing underlying functional deficits.

## Data Availability

Data supporting the findings of this study are available through the corresponding author, but restrictions apply to the availability of these data used for the current study and are therefore not publicly available. However, data are available from the corresponding author upon reasonable request.
